# The utility of the records medical: factors associated with the medication errors in chronic disease^1^

**DOI:** 10.1590/1518-8345.2406.2967

**Published:** 2017-12-11

**Authors:** Hellen Lilliane da Cruz, Flávia Karla da Cruz Mota, Lorena Ulhôa Araújo, Emerson Cotta Bodevan, Sérgio Ricardo Stuckert Seixas, Delba Fonseca Santos

**Affiliations:** 2 Graduated, Pharmacy, MSc, Departamento de Farmácia, Universidade Federal dos Vales Jequitinhonha e Mucuri, Diamantina, MG, Brazil.; 3 Especialist, Nursing and Family Health Especialization, Secretaria Municipal de Saúde, Prefeitura de Diamantina, Diamantina, MG, Brazil.; 4 Doctor, Pharmaceutical Sciences, Professor, Departamento de farmácia, Universidade Federal dos Vales Jequitinhonha e Mucuri, Diamantina, MG, Brazil.; 5 PhD, Estatistic, Professor, Departamento de Matemática, Universidade Federal dos Vales Jequitinhonha e Mucuri, Diamantina, MG, Brazil.; 6 PhD, Farmacology, Professor, Departamento de Farmácia, Universidade Federal dos Vales Jequitinhonha e Mucuri, Diamantina, MG, Brazil.; 7 PhD, Collective Health, Professor, Departamento de Farmácia, Universidade Federal dos Vales Jequitinhonha e Mucuri, Diamantina, MG, Brazil

**Keywords:** Medical Records, Primary Health Care, Medication Errors, Chronic Disease, Brazilian Public Health System

## Abstract

**Objective:**

This study describes the development of the medication history of the medical records to measure factors associated with medication errors among chronic diseases patients in Diamantina, Minas Gerais.

**Methods:**

retrospective, descriptive observational study of secondary data, through the review of medical records of hypertensive and diabetic patients, from March to October 2016.

**Results:**

The patients the mean age of patient was 62.1 ± 14.3 years. The number of basic nursing care (95.5%) prevailed and physician consultations were 82.6%. Polypharmacy was recorded in 54% of sample, and review of the medication lists by a pharmacist revealed that 67.0% drug included at least one risk. The most common risks were: drug-drug interaction (57.8%), renal risk (29.8%), risk of falling (12.9%) and duplicate therapies (11.9%). Factors associated with medications errors history were chronic diseases and polypharmacy, that persisted in multivariate analysis, with adjusted RP chronic diseases, diabetes RP 1.55 (95%IC 1.04-1.94), diabetes/hypertension RP 1.6 (95%CI 1.09-1.23) and polypharmacy RP 1.61 (95%IC 1.41-1.85), respectively.

**Conclusion:**

Medication errors are known to compromise patient safety. This has led to the suggestion that medication reconciliation an entry point into the systems health, ongoing care coordination and a person focused approach for people and their families.

## Introduction

Chronic disease (CD) is associated with significant morbidity and mortality, and constitutes a substantial burden on the health care system. This is especially true with systemic arterial hypertension and diabetes mellitus, which currently are the most common public health problems,^1^ and have a higher burden of disease in Brazil. 

Quality patient care is a priority issue in all health care sectors, however, medication errors (ME) are known to compromise patient safety^2^. A ME is any preventable event that may cause or lead to inappropriate medication use or patient harm; this has been studied extensively in developed countries^2^ ^-^ ^3^. A systematic review by Tam et al.^4^ identified 22 studies, involving a total of 3,755 patients, and found that errors in prescription medication histories occurred in more than 60% of cases. The most important finding of that study was an estimate that 59% of these errors had the potential to cause harm^4^.

Prevention of MEs has therefore become a high priority in patients with CD. Drug-related problems (DRP) may arise at all stages of the medication process, from prescription to treatment follow-up^5^. Therefore, medication reconciliation requires staff to: compile a full list of the patient’s previous medications, make a systematic comparison with the active prescriptions, and analyze and resolve any MEs^6^.

Pharmacists are increasingly being recognized as potential partners in many public health activities. Pharmacists have demonstrated their utility in many areas, including CD management^7^. The involvement of pharmacists in the medication use process, as members of the health care team, improves the quality of patient care by preventing MEs^8^ ^-^ ^9^. According to Winter,^8^ pharmacists are competent to supervise accurate medication histories and monitoring of error frequencies.

A particularly challenging field is the surveillance of pharmacotherapy in health service, which provides care for CD patients. Polypharmacy has been used in the context of prescribing or taking more medications that are clinically required^10^. Other authors divide the definition of polypharmacy into ‘appropriate’ and ‘problematic’ polypharmacy, which the authors of this paper believe supports distinguishing those patients who benefit from multiple medications and those who would benefit from review and reduction of multiple medications^11^. Similarly, health care delivery needs to be structured to improve patient outcomes^12^.

In recent years, the focus of research into optimization of medications for CD patients has shifted from quantitatively measuring the deficiencies in prescribing, to qualitatively uncovering the root causes of suboptimal prescribing^13^. From this research, new avenues for exploration have emerged that may optimize prescribing for CD patients, through targeted interventions and new procedures for medication reviews^13^. One of the most common recommendations after a medication review - discontinuation of medication, or deprescribing - is one of the least likely to be followed^9^. The deprescribing process includes some or all of the following elements: a review of current medications, identification of medications to be discontinued, a discontinuation regimen, involvement of patients, and a review with follow-up^14^.

This paper describes the development of the medication history of the medical records to measure factors associated with MEs.

## Methods

The subproject was part of a well-defined project entitled, “Risk stratification of hypertensive and diabetic patients from the perspective of the implementation and organization of care in the Viva Vida Integrated Center and Hiperdia Secondary Reference Center, in the Inter-municipal Consortium of Health, located in Alto do Jequitinhonha/MG”, developed in ten primary care units located in the city of Diamantina.

Ethical aspects included: the study was preceded by the approval of the Research Ethics Committee (CEP) of the Federal University of Jequitinhonha and Mucuri Vale (nº 1.460.253), and authorized by the directors of Municipal Health Secretary. The work was neither to discover nor to identify the professional that committed the error, but to analyze and detect the history of the ME. Therefore, the collected data was used exclusively by the researchers, guaranteeing the privacy of the information obtained.

Design, place and period: a retrospective, descriptive, observational study of association of secondary data, through the review of medical records of hypertensive and diabetic users, conducted in the municipality of Diamantina, Minas Gerais, from March to October of 2016.

Population and sample: according to the Basic Health Information System, a total of 5190 hypertensive and diabetic users were enrolled in 2015, constituting the population of the sample plan. From this screening, a total of 396 medical records were identified by simple sampling. Inclusion criteria were: diabetic and hypertensive patients, registered in primary care units in the year 2015, 18 years of age or older, with registration of the professional in the health unit between the years of 2013 and 2015. Exclusion criteria were: pregnant women, children, medical records without medications reported, last consult in a year prior to 2013.

Data collection instruments: the material used to evaluate the medication history was a structured form, divided into three parts. The first part included variables on the primary care service and socio-demographic variables, the second part described the primary health care, and the third part described the medications. A pilot test of the form was conducted with ten medical records, as a way to improve the collection instrument.

The medication list identified by the pharmacist was regarded as the most accurate list available in the medical record. The MEs were classified by reviewing the drug that may cause or lead to patient harm. The ME history included:

Polypharmacy: considered as the use of four or more medications ^15^. 

Medication-related problem: analysis of high risk medication^16^, low therapeutic margin^17^, renal risk^18^, inappropriate medication,^19^ and fall risk^20^.

Potential drug-drug interaction (DDI): identified and classified according to Micromedex,^21^ to determine potential moderate and major medication interactions among the 22 most common medical prescription. Micromedex was used to identify potential interactions among the list of common medications, and provided a measure of the severity of the interaction (contraindicated [the drugs are contraindicated for concurrent use]; major [the interaction may be life threatening, require medical intervention to minimize or prevent serious adverse events, or both]; moderate [the interaction may result in the exacerbation of the patient’s condition, require an alternation in therapy, or both]; and minor [the interaction would have limited clinical effects]). In our analysis, we focus on those drug-drug interactions considered to be potentially of moderate and major severity.

Duplicate therapy: simultaneous use of two medications from the same therapeutic subgroup, according to the Anatomical Therapeutic Classification (ATC), proposed by the World Health Organization^22^.

Data were analyzed using relative (percentage) and absolute (n) frequencies of the classes of each variable to characterize the sample studied. The quantitative variables were analyzed using means to summarize the information, along with standard deviations to indicate the variability of the data. Between-group differences were analyzed using a chi-square or Fisher’s exact tests, when appropriate. The multivariate analysis was based on Prevalence Ratio (PR), estimated by the Poisson regression model. We included in the initial Poisson model all the variables that, in the bivariate analysis, had an association with MEs at a level of significance less than 0.20. The significance level of 0.05 was the criterion adopted for maintaining the variable in the final Poisson model. The PRs with 95% confidence intervals (CI) were calculated.

## Results

The medical records of the hypertensive and diabetic patients totaled 396, representing 3.5% of the total family records. The mean age of patients was 62.1 ± 14.3 years, and the minimum and maximum ages of the patients were 25 and 100 years, respectively. Table 1 shows the characteristics of the medical records. In the primary health care, a higher percentage of those over 60 years of age, both men and women, was noted. Basic nursing skills were the most prevalent (95.5%), such as blood pressure measurement (302 or 76.3%), weight (259 or 76.3%), and height (233 or 58.8%). Physician consultations were performed in 82.6% of the patients, and among those physician specialists (39.9%), 8.3% were cardiologists.

Polypharmacy, a clinical characteristic, was recorded in 54% of the sample, with an average of 4.0 medications as noted in the medical records. The number of medications taken by patients ranged from 1 - 10. It was noted that 33.8% of the sample had comorbidities, of which 12.3% had heart disease, 10.9% obesity, 9.6% dyslipidemia, 6.1% cerebrovascular disease, and 2.3% depression. 

**Figure 1:**
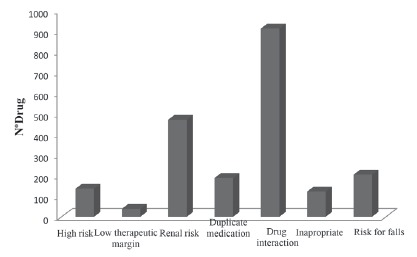
Distribution of medications with special characteristics registered in the medical records, Diamantina, MG, Brazil, 2016

A total number of 1577 medications were identified in the medical records. The patients on combination therapy were 14.2% more numerous than patients on monotherapy, and the most frequently used medication classes were: diuretics, angiotensin converting enzyme inhibitors, and beta blockers. Hydrochlorothiazide, captopril and propranolol were the most widely used agents representing these classes, respectively. The distribution of the medications with special characteristics registered is displayed in Figure 1. Review of the medication lists by a pharmacist revealed that 67.0% of the medications included at least one risk. The most common risks were: drug-to-drug interaction (57.8%), renal risk (29.8%), risk of falling (12.9%), and duplicate therapy (11.9%). The risk detected were distributed according to the specialties groups, the cardiovascular system (70.7%), alimentary tract and metabolism (15.8%), and the nervous system (6.9%).

**Table 1:** Demographics, care provided and clinical characteristics of the study population as found in the medical records, Diamantina, MG, Brazil, 2016

**Characteristics of the medical records**			
	**Hypertensive**	**Diabetic**	**Total**
**Characteristics of the patients**			
**Gender n (%)**			
**Female**	**229 (69.8)**	**47 (69.1)**	**276 (69.7)**
**Male**	**99 (30.2)**	**21 (30.9)**	**120 (30.3)**
Age groups n* (%)			
**18-29**	**5 (1.5)**	**0**	**5 (1.3)**
**30-39**	**19 (5.8)**	**4 (5.9)**	**23 (5.8)**
**40-49**	**38 (11.6)**	**3 (4.4)**	**41 (10.4)**
**50-59**	**90 (27.4)**	**12 (17.6)**	**102 (25.8)**
**≥60**	**176 (53.7)**	**49 (72.1)**	**225 (56.8)**
**Characteristics care provided**			
**Basic nursing care actions**	**314 (95.7)**	**64 (94.1)**	**378 (95.5)**
Home consultation (physicians or nurses) n* (%)	**43 (13.1)**	**10 (14.7)**	**53 (13.4)**
Physicians consultations in 12 months n*(%)	**272 (82.9)**	**55 (80.9)**	**327 (82.6)**
**Mean of consultations in 12 months ( ± s.d** ^**†**^ **)**	**2.7 (2.4)**	**3.1 (2.6)**	**2.8 (2.5)**
**Referral physician specialties**	**132 (40.2)**	**26 (38.2)**	**158 (39.9)**
**Physicians specialties**			
**Cardiologist**	**29 (22.0)**	**4 (15.4)**	**33 (8.3)**
**Orthopedist**	**30 (22.7)**	**3 (11.5)**	**33 (8.3)**
**Ophthalmologist**	**23 (17.4)**	**6 (23.1)**	**28 (7.1)**
**Neurologist**	**12 (9.1)**	**5 (19.2)**	**17 (4.3)**
**Angiologist**	**9 (6.8)**	**2 (7.7)**	**11 (2.8)**
**Urologist**	**9 (6.8)**	**0 (0)**	**9 (2.3)**
**Gynecologist**	**9 (6.8)**	**0 (0)**	**9 (2.3)**
**Otolaryngologist**	**5 (3.8)**	**3 (11.5)**	**8 (2.0)**
**Psychiatry**	**4 (3.0)**	**2 (7.7)**	**6 (1.5)**
**Endocrinologist**	**1 (0.8)**	**3 (11.5)**	**4 (1.0)**
**Dermatologist**	**4 (3.0)**	**0 (0)**	**4 (1.0)**
**Nephrologist**	**1 (0.8)**	**1 (3.8)**	**2 (0.5)**
**Pulmonologist**	**1 (0.8)**	**0 (0)**	**1 (0.3)**
**Rheumatologist**	**0 (0.0)**	**1 (3.8)**	**1 (0.3)**
**Clinical characteristics**			
**Poly-pharmacy n (%)**	**158 (48.2)**	**56 (82.4)**	**214 (54.0)**
**Mean medications (± s.d** ^**†**^ **)**	**3.7 (1.8)**	**5.5 (2.3)**	**4.0 (2.0)**
**Comorbidity n (%)**	**102 (31.1)**	**32 (47.1)**	**134 (33.8)**
**Mean comorbiditities (± s.d** ^**†**^ **)**	**0.4 (0.7)**	**0.7 (0.8)**	**0.4 (0.7)**

*n - number; †s.d - standard deviation

In addition to these, 76 (19.2%) medical records demonstrated that the patients were on duplicate therapy, as determined by review. Duplicate therapies included: glibenclamide/metformin, captopril/losartan, spironolactone/furosemide, and acetylsalicylic acid/clopidogrel. In the medication history, two medical records were found with contraindicated medication combinations: thioridazine/fluoxetine and metoclopramide/fluoxetine.

In this study, 65.9% of medical records presented drug-to-drug interactions (DDI). A total of 911 potential DDI were identified. Among these, 213 were classified as being potentially major severity, 489 were classified as potentially moderate severity, and 13 were classified as potentially minor severity. There was not one absolutely contraindicated DDI identified in the entire sample. 

Table 2 describes the DDIs found in the medical records, estimates the frequency of the use any prescription medications for those aged 50 to 59 years, and those 60 years and older. The medications that were most implicated in DDI were antihypertensives (80.1%) and antiplatelet agents (39.6%). In the older adults, the more common and potentially more significant DDI were those that affect renal function (49), reductions of blood pressure (36), nephrotoxicity (26), and hypoglycemia (21).

**Table 2:** Most frequent potential moderate and major medication interactions discovered in the medical records, Diamantina, MG, Brazil, 2015

**Drug interaction**	**Age 30-39y**		**Age 40-49y**		**Age 50-59y**		**Age ≥60y**		**Total**	**Potential interaction effect**
	**Female**	**Male**	**Female**	**Male**	**Female**	**Male**	**Female**	**Male**		
**Moderate**										
**Captopril /Hydrochlorothiazide -**	**1**	**2**	**7**	**0**	**14**	**6**	**17**	**15**	**62**	**Reduction of blood pressure**
**Losartan/Salicylic acid**	**0**	**0**	**2**	**3**	**5**	**1**	**15**	**9**	**35**	**Renal dysfunction**
**Enalapril/Hydrochlorothiazide**	**2**	**0**	**5**	**2**	**9**	**3**	**10**	**4**	**35**	**Reduction of blood pressure**
**Hydrochlorothiazide /Propranolol**	**0**	**0**	**4**	**1**	**9**	**3**	**8**	**6**	**31**	**Hyperglycemia**
**Salicylic acid/Enalapril**	**0**	**0**	**2**	**1**	**5**	**3**	**7**	**7**	**25**	**Renal dysfunction**
**Salicylic acid/Atenolol**	**1**	**0**	**0**	**2**	**4**	**1**	**9**	**5**	**22**	**Increased blood pressure**
**Salicylic acid/ Captopril**	**0**	**0**	**1**	**1**	**3**	**3**	**6**	**5**	**19**	**Renal dysfunction**
**Clopidogrel/Simvastatin**	**0**	**0**	**1**	**0**	**3**	**2**	**7**	**3**	**16**	**High platelet reactivity**
**Insulin/ Metformin**	**0**	**1**	**0**	**0**	**0**	**0**	**8**	**3**	**12**	**Hypoglycemia**
**Insulin/Losartan**	**0**	**0**	**0**	**0**	**0**	**1**	**8**	**2**	**11**	**Hypoglycemia**
**Levothyroxine/Sinvastatim**	**0**	**0**	**0**	**0**	**2**	**1**	**6**	**2**	**11**	**Decreased levothyroxine efficacy**
**Major**										
**Salicylic Acid /Hydrochlorothiazide**	**1**	**0**	**2**	**3**	**7**	**1**	**18**	**8**	**40**	**Possible nephrotoxicity**
**Furosemide/ Salicylic acid**	**0**	**0**	**1**	**1**	**1**	**1**	**9**	**9**	**22**	**Reduced diuretic**
**Anlodipine/Simvastatin**	**0**	**0**	**1**	**0**	**2**	**1**	**10**	**4**	**18**	**Rhabdomyolysis**
**Clopidogrel/ Salicylic acid**	**0**	**0**	**1**	**0**	**3**	**2**	**5**	**2**	**13**	**Increased risk of bleeding**
**Spironolactone/ Salicylic acid**	**0**	**0**	**0**	**0**	**0**	**0**	**7**	**1**	**8**	**Reduced diuretic**
**Captopril/Losartan**	**0**	**1**	**1**	**1**	**1**	**2**	**1**	**1**	**8**	**Increased risk of adverse events**
**Simvastatin/carbamazepine**	**0**	**0**	**1**	**1**	**0**	**0**	**1**	**3**	**6**	**Reduced simvastatin exposure**
**Salicylic acid/Digoxin**	**0**	**0**	**0**	**0**	**0**	**0**	**3**	**3**	**6**	**Prolonged half-live of digoxin**
**Enalapril/Allopurinol**	**0**	**1**	**0**	**0**	**2**	**1**	**1**	**2**	**6**	**Hypersensitivity reactions**
**Fluoxetine/ Salicylic acid**	**0**	**0**	**1**	**0**	**1**	**0**	**3**	**0**	**5**	**Risck of bleeding**
**Warfarin /Simvastatin**	**0**	**0**	**0**	**0**	**0**	**0**	**3**	**1**	**4**	**Rhabdomyolysis**

In the univariate analysis, MEs were associated with these variables: age (p=0.0002), CD (p<0.0001), cerebrovascular disease comorbidity (p=0.0090), and polypharmacy (p<0,0001) but not with sex or number of physician specialties (Table 3). 

**Table 3:** Comparison between medication errors and characteristics described in the medical records, Diamantina, MG, Brazil, 2016

	**Medication error history (%)**		**p-value**
	**Yes (n = 300)**	**No (n = 96)**	
**Gender n (%)**			0.3595*
**Female**	**68.3**	**74.0**	
**Male**	**31.7**	**26.0**	
**Age groups n (%)**			**0.0002** ^**†**^
**18-29**	**4.2**	**0.3**	
**30-39**	**9.4**	**4.7**	
**40-49**	**18.8**	**7.7**	
**50-59**	**21.9**	**27.0**	
**≥60**	**45.7**	**60.3**	
**Chronic disease**			**< 0.0001** ^**†**^
**Hypertension**	**77.3**	**100.0**	
**Diabetes**	**2.7**	**0**	
**Diabetes/Hypertension**	**20.0**	**0**	
**Comorbidity**			
**Cardiovascular diseases**	**16.7**	**9.4**	0.1137*
**Dyslipidemia**	**10.3**	**7.3**	0.4955*
**Cerebrovascular diseases**	**8.0**	**0.0**	0.0090*
**Obesity**	**11.0**	**7.3**	0.3926*
**Depression**	**2.7**	**1.0**	**0.6938** ^**†**^
**Polypharmacy**			< 0.0001*
**Yes**	**67.0**	**13.5**	
**No**	**33.0**	**86.5**	
**Physician specialty**			
**Cardiologist**	**8.3**	**8.3**	1.0*
**Orthopedist**	**7.7**	**10.4**	0.5245*
**Ophthalmologist**	**7.3**	**6.2**	0.8952*
**Neurologist**	**4.7**	**3.1**	**0.7727** ^**†**^
**Angiologist**	**3.3**	**1.0**	**0.3090** ^**†**^
**Urologist**	**2.7**	**1.0**	**0.6938** ^**†**^
**Gynecologist**	**2.3**	**2.1**	**1.0** ^**†**^

*Pearson’s chi-square test (significant if p <0.05). †Fisher’s exact test (significant if p <0.05); n: number.

Factors associated with ME history in the multivariate analysis are presented in Table 4. Interestingly, association observed between MEs, CD and polypharmacy persisted in the multivariate analysis, with adjusted PR CD, diabetes PR 1.55 (95%IC 1.04-1.94), diabetes/hypertension PR 1.6 (95%CI 1.09-1.23) and polypharmacy PR 1.61 (95%IC 1.41-1.85), respectively. 

**Table 4:** Model of the multivariate analysis to predict medication error history outcome, Diamantina, MG, Brasil, 2016

**Variables**	**Medication errors history (%)**	PR*	**CI† (95%)**	**p-value**
**Ages**				
**18-29**	**20.0**	**1.00**	**---**	**---**
**30-39**	**60.9**	**2.53**	**(0.51, 12.45)**	**0.2534**
**40-49**	**56.1**	**2.51**	**(0.52, 12.19)**	**0.2551**
**50-59**	**79.4**	**3.27**	**(0.68, 15.74)**	**0.1386**
**≥60**	**80.4**	**3.08**	**(0.64, 14.79)**	**0.1606**
**Chronic disease**				
**Hypertension**	**70.7**	**1.00**	**---**	**---**
**Diabetes**	**100.0**	**1.55**	**(1.24, 1.94)**	**0.0001**
**Diabetes/Hypertension**	**100.0**	**1.16**	**(1.09, 1.23)**	**< 0.0001**
**Comorbidity**				
**Cardiovascular diseases**				
**Yes**	**84.7**	**1.08**	**(0.97, 1.21)**	**0.1648**
**No**	**74.2**	**1.00**	**---**	**---**
**Cerebrovascular diseases**				
**Yes**	**100.0**	**1.08**	**(0.99, 1.18)**	**0.0765**
**No**	**74.2**	**1.00**	**---**	**---**
**Polypharmacy**				
**Yes**	**93.9**	**1.61**	**(1.41, 1.85)**	**< 0.0001**
**No**	**54.4**	**1.00**	**---**	**---**

*Poisson regression (significant if p <0.05). Only the independent variables that obtained p-value <0.20, in the univariate analysis, were included in the multivariate model; *PR: Prevalence ratio; †CI: confidence intervals

## Discussion

This study demonstrates that a medical record provides the use of medications by diabetic and hypertensive patients, and can be used to assess the impact of primary care management. It can also be used to assess the application of the structured medication history use tool to optimize prescribing, and to reduce MEs. The findings from this study are as follows:

(1) Within the medication history obtained by the medical record, a history of MEs was found in the majority of patients (75.7%). Our findings are higher than those of other studies^4^ ^,^ ^8^.
(2) The majority (35.4%) of the medications are involved with potential DDI. The possibility of DDI (66.2%) detected was higher than that found by other authors^23^ (16.3%). This can be attributed to the fact that cardiovascular drugs are the most common drugs to cause DRP^4^ ^,^ ^24^.
(3) A percentage of medications with renal risk were found (29.8%). This result is probably due to the fact that the study group uses medications with active ingredients that cause nephrotoxicity.
(4) The frequency of therapeutic duplicity in this study was lower than 70.0%,^25^ and higher 7.6%^26^.

The large difference in percentage of MEs in the medical records as a result is interesting. The drugs most involved in medication errors, according to the ATC anatomic group were those related to the cardiovascular system, alimentary tract and metabolism and nervous system. A systematic review^4^ stated that the prescription drugs most often involved in ME history are cardiovascular agents and sedatives. Another study found mainly antihypertensives were involved^27^.

In absolute numbers, this study found the procedures most recorded were basic nursing care activities (95.5%), and this information is useful to assess the profile of work in primary care, characterized by preventive and curative actions. This profile is different from that described by other authors^28^ (33.0%), who compared 240 primary care units from seven southern and northeastern states. This may reflect the demographic profile and needs, depending on the region. Another important finding is the attendance of medical specialties in 39.9% of the sample. These are similar to those when studying the quality of basic care in covered areas^29^.

The percentage of medical care present in this study can be used as an indicator of the ability to determine the medication history. This was also discussed by other authors^30^ who concluded that the frequency of medication histories taken by physicians is significantly influenced by their specialties. Patients in these specialties are often diagnosed with two or more comorbidities requiring multiple medication therapy^30^. For example, hypertensive patients often have coexisting diabetes, coronary artery disease, or other cardiovascular disorders. This may well explain the percentage of the medication history related to prescription drugs for CD^31^.

Literature on health care service use in Brazil^28^ ^-^ ^29^ ^,^ ^32^ has found that the nursing care and physical examination stand out, as in our study. However, there are gaps in the research regarding scientific evidence of medication history, polypharmacy, and DDI in patients with comorbidities.

The majority of DDI in our study were of a moderate severity (53.7%). The most common potential DDI in this group was the interaction between angiotensin-converting enzyme inhibitors and loop diuretics (captorpil/hydrochlortiazide), as noted in another study^33^. Also in this study, 23.4% of the patients were exposed to potentially severe medication combinations. In the literature, the prevalence of potential DDI in community-dwelling patients ranges from 4 - 46%^23^ ^-^ ^24^ ^,^ ^34^. According to the data, acetylsalicylic acid (25.1%) was the drug that caused such interactions in hypertensive and diabetic patients in another study ^24^ ^)^ (28.0%), higher than the 5.3% noted in a different study^33^.

The other aspect of the study was the association between persistent MEs after adjusting for variables, including CDs and polypharmacy. Many studies have described ME rates in hospital settings, but data for primary care is relatively scarce^35^ ^).^ The MEs have been well studied within the context of the health care system, and nearly 40% of errors originate with prescribing^36^. One of the biggest challenges in preventing ME and polypharmacy are the substantial gap between theory and clinical practice. These results suggest that more caution should be taken in the monitoring of polypharmacy MEs. In 11% of patients experiencing a ME, risk factors included: poor coordination of care, cost-related barriers to prescription medications costs, multimorbidity, and hospitalization^3^.

There are several potential solutions to reducing MEs and improving medication safety. Importantly, most interventions have been conducted in the different levels of services available. Strategies employed include using the recently published National Institute for Health and Care Excellence (NICE) guideline on multimorbidity and drug optimization^37^ ^-^ ^38^, and to develop and agree upon an action plan for multimorbidity and polypharmacy to inform medication optimization. Among these is supporting clinicians in developing an individualized, patient-centered approach to reviewing patients with multimorbidity and polypharmacy^37^ ^-^ ^38^. 

On the other hand, much attention has recently focused on primary care services as the heart of integrated people-centered health care^35^. According to data from one systematic review of 38 studies of primary care interventions, that most successful intervention included a medication review conducted by a pharmacist, leading to a reduction in hospital admissions^39^. Based on data in the literature^35^, we consider that continuity of clinical ME management would be the most appropriate use of medication reconciliation. Consequently, new medication changes, deletions, and additions can be monitored in the primary care.

This study offered support to develop methods for the predictive modeling of health outcomes in pharmacovigilant activities. The survey also highlighted opportunities, such as: medication reconciliation as an entry point into the health systems, ongoing care coordination, and a person-focused approach for patients and their families. 

Unfortunately, an accessible and complete medication list is not available. This is true for Brazil, where medication histories are still issued in a paper-based format. However robust research is needed to assess the impact of medication history. In addition, electronic-SUS(E-SUS) and the Integrated Management System for Pharmaceutical Assistance (SIGAF) programs may confer additional benefits for patients in the future, such as improvements in patient safety and increased involvement, through routine monthly consultations with pharmacists.

There were a few potential limitations to this study. First, because of the complexity of the medication process and its associated, multifaceted factors, there may have been many other contributing factors to errors that we could not observe or understand. The second of the possible limitations was a study design that was restricted to a quantitative study of the ME history; however a qualitative evaluation of the potential consequences caused by the ME history would have greater clinical relevance. A qualitative investigation, however, was not a pre-defined endpoint of our study. Potential harm caused by ME history, and consequent medication reconciliation, can only be evaluated in cohort observational study (without reporting of the pharmacist-acquired medication histories) or a randomized trial (pharmacist- versus physician-acquired medication histories). A complete medication history is very time-consuming, and can conceal a medication-related problem. 

## Conclusion

The occurrence of ME in the municipality of Diamantina is a common condition among patients with CD, as is the use of polypharmacy in primary health care. Despite the limitations of the study, it should be highlighted that these factors certainly need to be individually treated in all health care services. In this context, knowing the medication history is important, so that medication reconciliation occurs at the points of entry into the health system.
